# Factors Predicting Self-Care Behaviors among Low Health Literacy Hypertensive Patients Based on Health Belief Model in Bushehr District, South of Iran

**DOI:** 10.1155/2018/9752736

**Published:** 2018-02-13

**Authors:** Azam Larki, Rahim Tahmasebi, Mahnoush Reisi

**Affiliations:** ^1^Department of Health Education and Health Promotion, Bushehr University of Medical Sciences, Bushehr, Iran; ^2^Department of Biostatistics, Bushehr University of Medical Sciences, Bushehr, Iran

## Abstract

The aim of this study was to determine the factors influencing adherence to self-care behaviors among low health literacy hypertensive patients based on health belief model. A cross-sectional study was conducted among 152 hypertensive patients with low health literacy. Patients with limited health literacy were identified by S-TOFHLA. The data were collected using H-scale for assessing self-care behaviors and, HK-LS for assessing knowledge of hypertension. A researcher-made questionnaire was applied for collecting data of health belief model constructs. Data were analyzed by SPSS version 22 with using multiple logistic regression analyses. Perceived self-efficacy was associated with all self-care behaviors except medication regimens. There was a significant association between perceived susceptibility and adherence to both low-salt diet (OR = 3.47) and nonsmoking behavior (OR = 1.10). Individuals who had more perceived severity (OR = 1.82) had significantly greater adherence to their medication regimens. Perceived benefits and barriers were not significantly associated with either type of hypertension self-care behaviors. It seems that designing and implementation of educational programs to increase self-efficacy of patients and promote their beliefs about perceived susceptibility and severity of complications may improve self-care behaviors among low health literacy hypertensive patients.

## 1. Introduction

Hypertension is an important worldwide public-health challenge, which can lead to very serious consequences such as cardiovascular and kidney disease [1]. According to reports, more than one in three adults worldwide suffer from high blood pressure, and this proportion increases with age [2]. Due to the high prevalence of hypertension and its serious complications, the World Health Organization (WHO) assigned the theme of World Health Day 2013 to hypertension as a “silent killer, global public-health crisis” [3]. Statistics showed an increase in the prevalence of hypertension. The number of patients with hypertension has increased from 600 million cases in 1980 to 1 billion in 2008; over 40 percent of adults were known to have hypertension [4]. Also, it was predicted that, by 2025, 1.56 billion adults will suffer from hypertension [5]. In this regard, it was reported to be 14 to 34 percent hypertensive patients in Iran [6].

Although this disease can lead to acute and debilitating complications and imposes many costs on the individual and the healthcare system, studies show that blood pressure control in hypertensive patients is not desirable, as the results of various studies all over the world suggest a high prevalence of uncontrolled blood pressure among people with hypertension [7, 8].

The most important strategy for controlling blood pressure and maintaining it in the optimal range is patient compliance with self-care behaviors [9]. The findings of a meta-analysis that examined the results of 87 studies indicated that optimal self-care in hypertensive patients could reduce systolic and diastolic blood pressure by 5 and 4.3 mmHg, respectively [10]. Self-care for people with high blood pressure includes compliance with a healthy diet (especially low salt), physical activity, nonsmoking, abstaining from alcohol, weight management, and following prescribed medications [11]. Despite the necessity of carrying out all self-care behaviors to succeed in the management of hypertension, globally, many patients do not follow medical or lifestyle recommendations and therefore compliance with self-care behaviors in these patients is not desirable [12].

Based on evidence, self-care behaviors and blood pressure control become worse when hypertensive patients have limited health literacy [13]. Low health literacy may impact the ability to perform tasks such as understanding basic written health information or reading a prescription correctly. Low health literacy also is associated with worse chronic disease control, increased utilization of the emergency department and hospital care, and increased mortality. Lower health literacy has been associated with worse hypertension-related knowledge, lower ability to identify hypertension medications, and reduced adherence to cardiovascular medication refills. Various studies suggest that self-care behaviors are influenced by demographic variables and modifiable psychological variables [11, 14, 15]. Information about factors that affect self-care behaviors in low health literacy hypertensive patients based on theoretical framework is scarce. In this study, the HBM (Health Belief Model) was used to explain factors related to self-care behaviors of low health literacy hypertensive patients. HBM is one of the most important theories of behavior change that has been widely considered in behavioral health sciences and successfully applied in the design of health interventions. This model has emphasized the role of moderating factors (demographic, social, and structural factors) and individual perceptions (perceived sensitivity, perceived severity, perceived benefits, perceived barriers, guidance for action, and self-efficacy) in determining the likelihood of performing a behavior [16]. According to this model, a person's decision and motivation to perform a particular behavior included items such as person's perception of being at risk (perceived susceptibility) and its seriousness (perceived severity), belief in the perceived action of usefulness to reduce the risk of disease, understanding of the health benefits (perceived benefits), person's perception of the difficulties and cost of performing behaviors (perceived barrier), and moderating factors such as demographic and psychosocial variables (awareness) and people's judgments of their capabilities to execute given level of performance (self-efficacy) [17].

Therefore, the purpose of this study was to determine the factors related to self-care behaviors among low health literacy hypertensive patients based on health belief model. The results can be used as baseline data to improve self-care behaviors and blood pressure control caused by psychological factors.

## 2. Material and Methods

This cross-sectional study was conducted on 152 patients with limited health literacy who had been referred to the Haft-e-Tir Comprehensive Health Service Center in Bushehr city. This study was approved by the Ethics Committee of Bushehr University of Medical Sciences (IR.BPUMS.REC.1395.128). At first, patients were identified based on initial entry criteria and entered the study by convenience sampling method. Initial entry criteria for participation in the study include having appropriate physical conditions for answering questions, age over 30 years, not having a serious complication due to hypertension (diseases such as cardiovascular disease, neuropathy, nephropathy, retinopathy, and stroke), and passing at least 6 months of definite diagnosis of the disease. The final entry criteria for the study were limited health literacy. To identify patients with limited health literacy, a Short version of Test of Functional Health Literacy in Adults (S-TOFHLA) was completed. After identifying patients with limited health literacy and before the interview, the interviewer first explained the purpose of the survey, the study participants' rights, the risk and benefit of participation, and the plan to protect the confidentiality of study participants. Further, a signed informed consent was obtained prior to the interview. Then for patients who had proclaimed their consent to participate in the study, health beliefs model and self-care behaviors questionnaire were completed. Of the 209 participants identified on the basis of initial entry criteria and convenience sampling method, 152 had limited health literacy and completed the other questionnaires.

### 2.1. Measurement

#### 2.1.1. Health Literacy

Health literacy was evaluated by a shortened version of the Test of Functional Health Literacy in Adults (S-TOFHLA) that included two reading passages (36 items worth 2 points each) and 4 numeracy items (7 points each) to assess comprehension of hospital forms and labeled prescription vials that contained numerical information; this test also assesses quantitative skills and the ability to read and understand prose and documents. Possible scores on the S-TOFHLA range from 0 to 36. Based on the cut of points of the questionnaire, we categorized patients into three mutually exclusive groups: inadequate, marginal, or adequate health literacy. Scores from 0 to 55 indicate inadequate health literacy. Scores from 56 to 66 indicate marginal health literacy, and scores from 67 to 100 indicate adequate health literacy. The Persian version of the scale shows adequate internal reliability for numeracy (Cronbach's *α* = 0.69) and for reading comprehension (Cronbach's *α* = 0.78) [18].

#### 2.1.2. Knowledge of Hypertension

Hypertension knowledge assessed using Hypertension Knowledge Level Scale (HK-LS). This 22-item scale prepared by Erkoc et al. [19]. HK-LS assesses respondents' knowledge in defining hypertension, lifestyle, medical treatment, drug compliance, diet, and complications of hypertension. Each item is a full sentence that is either correct or incorrect. And each item is prepared as part of a standard answer (correct, incorrect, or do not know). Zinat Motlagh et al. have validated this questionnaire in Iranian population [20]. In Persian version in the validation process, three items were excluded from the scale and the final version has 19 true/false items.

#### 2.1.3. Health Belief Model Constructs

In order to assess the constructs of health belief model, a researcher-made questionnaire was used. Items developed for susceptibility, seriousness, benefits, barriers, and self-efficacy focused on self-care behaviors in hypertensive patients. 39 items with 5-point Likert answers was used (9 items for perceived benefits, 7 items for perceived barriers, 9 items for perceived susceptibility, 6 items for perceived severity, and 10 items for perceived self-efficacy). For determination of content validity, the list items were distributed to judges who were faculty members and Ph.D. candidates and they were quite familiar with HBM constructs. In content validity, changing the format of questions and omitting irrelevant questions were performed. Then, mean Content Validity Index (CVI) and Content Validity Rate (CVR) of the questionnaire were calculated as 0.94 and 0.91, respectively. Reliability of the scale was calculated and Cronbach's alpha values were 0.71, 0.70, 0.70, 0.82, and 0.85 for perceived susceptibility, perceived severity, perceived barriers, perceived benefits, and perceived self-efficacy, respectively.

#### 2.1.4. Self-Care Behavior

Self-care behaviors were determined using the hypertension self-care activity level effects (H-scale). This is a 31-item scale and was prepared by Zinat Motlagh [20]. The H-scale is designed to help primary care physicians to guide hypertensive patients who are seeking to achieve blood pressure control [21]. The H-scale examines the level of self-care by asking about the number of days per week on which an individual performs a self-care behavior. The H-scale was previously validated in Persian patients with high blood pressure [20]. The Persian version consisted of 27 items that measures the hypertension self-care activities with the following domains: medication adherence (3 items), physical activity (2 items), low-salt diet (10 items), smoking (2 items), alcohol (1 item), and weight management (9 items). The Persian version of the scale shows adequate internal consistency. Cronbach alphas were as follows: medication adherence (Cronbach's *α* = 0.91), low-salt diet (Cronbach's *α* = 0.72), physical activity (Cronbach's *α* = 0.96), smoking (Cronbach's *α* = 0.91), and weight management (Cronbach's *α* = 0.85).

#### 2.1.5. Sociodemographic Characteristics

Sociodemographic attributes, including age, sex, marital status, education level, and hypertension duration, were collected. Levels of education were divided into four categories: (1) illiterate; (2) primary school (1–5 years of schooling); (3) secondary/high schooling (6–12 years of schooling); and (4) education above high school. The number of years between the diagnosis of hypertension and point of data collection were obtained as disease duration.

### 2.2. Data Analysis

The data obtained from the total of 152 completed questionnaires in hypertensive patients with limited health literacy. Descriptive statistics were used to examine the characteristics of the sample. Then the participants for each of self-care behaviors were divided into two groups. First group is the low literacy patients who reported to perform self-care behaviors and second group is the low literacy patients who did not perform self-care behaviors. The multiple logistic regression analyses with forward steps likelihood ratio method was used to determine of predictive HBM constructs. All statistical analyses were performed using Statistical Package for Social Sciences (SPSS) version 22.0. In all tests, the level of significance was 0.05.

## 3. Results

### 3.1. Sample Characteristics

A total of 152 hypertensive patients with limited health literacy were studied. Their demographic features are shown in Table 1. The mean age of participants was 56.87 ± 8.70 ranging from 35 to 80 years (Table 1). Almost, 42 participants were male (27.6%) and 110 were female (72.4%). Participants had an average of 4.46 ± 5.8 years of diagnosed hypertension. Most of the subjects (84.9%) were married and had secondary/high school education (48.7%). More than half of patients (57.9%) reported earning more than 10 million IR-Rial (≥250$) a month. Most of the patients (61.8%) did not work nor were employed at the time of the study. Family history of hypertension was reported in 48.7% of patients. 34.9% of patients had their systolic hypertension above 140 and diastolic blood pressure above 90 in their last care at the health center. The most important sources for receiving health information were health professionals (42.1%) and then Internet based messaging software such as Telegram and WhatsApp (17.8).

Our results revealed that only 9.2% had a proper adherence to their medication regimen, and 5.3% avoided salt both while cooking and eating. Moreover, 19.1% had physical activity on most weekdays, and 55.9% were nonsmokers. 21.1% were alcohol consumers, and 27% managed their weight (Table 2).

The means and standard deviations of knowledge 7.75 (SD = 2.89) and HBM constructs related to self-care behaviors were 38.60 (SD = 7.87) for self-efficacy, 16.78 (SD = 4.01) for perceived susceptibility, 13.36 (SD = 3.35) for perceived severity, 32.90 (SD = 7.07) for perceived benefits, and 14.92 (SD = 4.70) for perceived barriers.

The multiple logistic regression analysis was used to assess the predictors of adhering self-care behaviors for HBM constructs. The results of the multiple logistic regression indicated that the knowledge about hypertension and self-care was predictor of weight management (OR = 1.247) and medication regimens (OR = 1.376), that is, the patients who had known about hypertension and self-care behaviors were one time more likely to managed their weight and more likely to adhere to their medication regimen than those who had not. Self-efficacy was predictor of all self-care behaviors except no alcohol and medication regimens. Patients with greater perceived self-efficacy were more likely to have weight management (OR = 1.174) and to be nonsmokers (OR = 1.069), physically active (OR = 1.092), and to adhere to a low-salt diet (OR = 1.259) than those with lower self-efficacy. Perceived susceptibility about hypertension complications and adherence was predictor of both low-salt diet (OR = 1.139) and nonalcohol behavior (OR = 1.562). The results also revealed that individuals who had more perceived severity (OR = 1.301) had significantly greater adherence to their medication regimens compared to those who had less perceived severity. The other components of HBM (perceived benefits and barriers) were not significantly associated with either type of hypertension self-care behaviors (Table 3) and none of the HBM components predict alcohol consumption among participants.

## 4. Discussion

Blood pressure control in hypertensive patients was considered as a long-standing challenge. This can be more challenging, when hypertensive patients have limited health literacy. Therefore, the aim of this study was to determine the factors related to self-care behaviors as the most important way to control high blood pressure among low health literacy hypertensive patients based on health belief model. Based on findings, low health literacy hypertensive patient's adherence to self-care tasks was low in terms of healthful diet, physical activity, weight management, and medication adherence and was moderate regarding nonsmoking and alcohol abstinence.

Based on our primary results more than 90% of the participants had not a low-salt diet and they reported that they added extra salt to their food while cooking and eating. This is while the WHO suggests that every adult should consume less than 5 grams of sodium each day [22]. However, in Iran and most countries, the daily consumption of salt per person is between 9–12 grams on average [23]. This well has been confirmed that the consumption of foods containing salt accompanied by the insufficient consumption of fruits and vegetables is an important factor that lead to high blood pressure and unsuccessful management of this disease. Thus, the implementation of health interventions to reduce the consumption of salt expenditure, as well as increasing the consumption of fruits and vegetables, is a fundamental health need.

Other results show that less than one-fourth (19.1%) of the participants took part in physical activity at least 30 minutes almost every day and only 27% of the sample managed their weight. Similar to our results the other study found that 81.2% of people with arterial hypertension did not perform any kind of physical activity [24]. This is despite the fact that the WHO has suggested 30 minutes of physical activity five days a week to prevent and control high blood pressure [25]. The amount of physical activity in the study population is much lower than what has been reported by WHO. Insufficient physical activity and lack of weight management are topics that need more attention from physicians and healthcare providers but instead seems to have been ignored by them.

The prevalence of adherence to medication as the other self-care behaviors was low in study population. The overall adherence to medication in our study was 9.2% as compared to a similar study; the adherence was 48.7% [26] and, in an Iranian study by Kamran et al., 24% of the patients were adherent [14]. Probably the reason of this difference is due to limited health literacy of this study's population. It seems that adherence to medication regimen is less important for limited health literacy patients with hypertension. Therefore, the implementation of educational interventions to inform patients with hypertension about the necessity of antihypertensive drugs are essential.

We found a moderate rate of smoking and alcohol consumption in study population. Most of patients in this study avoided tobacco use (55.9%) and did not consume alcohol (78.9%). Despite the fact that smoking and alcohol consumption in Iran are not socially and culturally accepted, and, even being illegal in case of alcohol, we still see the consumption of these substances in individuals and even in patients. Multiple factors contribute to these behaviors. It is possible that participants do not associate reducing smoking and alcohol consumption as hypertension self-care behaviors. This reasoning would suggest that health providers should intervene to increase awareness of alcohol consumption and its effects on hypertension management.

The results indicated that knowledge about hypertension and self-care was significantly associated with weight management and medication regimens. This finding declares the role of knowledge as a major source and confirm a large body of research [27] that suggests knowledge about hypertension and self-care can enhance the ability to cope and comply with medical regimens and disease management.

Analyzing Health Belief Model, our study found significant associations among HBM components. The findings indicated that self-efficacy is associated with adhering to low-salt diet, engaging in physical activity, not smoking, and utilizing common weight management strategies in hypertensive patients with low literacy. Our finding supports a positive relationship between self-efficacy and self-care behaviors found in previous studies [11, 28, 29]. Self-efficacy plays a crucial role in adoption of hypertension-controlling behaviors [30] and, as mentioned in other studies [31], it seems that patients with low health literacy may feel less confident in their ability to perform self-care behaviors and may have less motivation for performing self-care tasks. Consequently, increased confidence of low health literacy patients with regard to selecting appropriate behaviors seems to improve adherence to self-care tasks. Therefore, it is useful that through educational interventions, educators promote the patient's ability to perform self-care behaviors through self-efficacy strategies.

Our results showed that perceptions of susceptibility were influential for low-salt diet and nonsmoking behavior. These self-care behaviors were worse among those who had lower perception regarding the susceptibility or vulnerability to the disease process. Perceived susceptibility is one of the most important factors affecting health behaviors. Dehghani-Tafti et al. [29] introduced perceived susceptibility as a key factor in behavioral changes among diabetic patients. For the hypertensive patients with low health literacy to adopt self-care behaviors, health providers should intervene to change their beliefs to the point that they are susceptible and at risk for the complications of the disease.

Similar to other studies, medication adherence decreased with lower perception of disease severity [14, 32]. This may be due to poor health literacy that which makes the patients to have less knowledge about hypertension and its complications among these patients. Therefore, it seems that educating patients with limited health literacy about blood pressure and its complications can be effective in creating beliefs to understand the threat of high blood pressure.

In conclusion based on findings, there was minor adherence for self-care behaviors among patients with high blood pressure and low health literacy. This is due to the inadequate knowledge, perceived susceptibility, perceived severity, and perceived self-efficacy. Therefore, it is necessary that health providers improve their actions and also their communications with the patients especially patients with low health literacy to ensure a better influence on self-care behaviors. Health education based on HBM components can be useful and educational programs for these vulnerable patients should also be expanded.

Since this study was based on a convenience sample, the findings of this study may not be generalized to all Iranian hypertensive patients with low health literacy. Self-care behaviors were evaluated with self-report, so the reliability of self-rated self-care behaviors, in particular, may be subject to recall bias or memory failure. The results reported in the study were obtained from a cross-sectional survey and no causality is established between HBM components and self-care tasks. A longitudinal study that follows the study sample and reassesses their health outcomes at a later time would help to discern the causal effects of HBM components. The S-TOFHLA, similar to other health literacy assessments, offers no indication about the respondent's communication skills, which may be equally important in determining an individual's ability to effectively navigate today's complex health care system.

## Figures and Tables

**Table 1:** The characteristics of respondents and descriptive findings (*n* = 152).

Variables	
*Age (years)*	Mean age: 56.87 years (SD = 8.70)
	Range: 35–80 years
*Years with hypertension*	Mean: 9.46 years (SD = 5.80)
*Gender*	Range: 1–30 years
Male	42 (27.6%)
Female	110 (72.4%)
*Marital status*	
Never married	3 (2%)
Married	129 (84.9%)
Divorced	20 (13.9%)
*Educational level*	
Illiterate	5 (3.3%)
Primary school	61 (40.1%)
Secondary/high school	77 (48.7%)
Above high school	9 (5.9%)
*Family history of hypertension*	
Yes	74 (48.7%)
No	78 (51.3%)
*Income*	
<10000000 IR-Rial	64 (42.1%)
>10000000 IR-Rial	88 (57.9%)
*Medication *	
Yes	149 (98%)
No	3 (2%)

**Table 2:** Self-care behaviors frequencies and percentages.

Self-care behavior	Number (%)
Medication adherence	14 (9.2)
Eating a low-salt diet	8 (5.3)
Physical activity	29 (19.1)
Nonsmoking	85 (55.9)
Alcohol abstinence	120 (78.9)
Weight management	41 (27.0)

**Table 3:** Associations between knowledge, structures, Health Belief Model, and hypertension self-care behaviors.

	Weight management OR (95% CI)	Nonalcohol OR (95% CI)	Nonsmoking OR (95% CI)	Physical activity OR (95% CI)	Low-salt diet OR (95% CI)	Medication regimens OR (95% CI)
Knowledge	1.247 (1.064–1.461)					1.376 (1.080–1.753)
Perceived susceptibility		1.139 (1.024–1.267)			1.562 (1.031–2.366)	
Perceived severity						1.301 (1.040–1.626)
Perceived self-efficacy	1.174 (1.092–1.261)		1.069 (1.044–1.149)	1.092 (1.031–1.157)	1.259 (1.053–1.505)	
